# Walnut consumption in a weight reduction intervention: effects on body weight, biological measures, blood pressure and satiety

**DOI:** 10.1186/s12937-017-0304-z

**Published:** 2017-12-04

**Authors:** Cheryl L. Rock, Shirley W. Flatt, Hava-Shoshana Barkai, Bilge Pakiz, Dennis D. Heath

**Affiliations:** Department of Family Medicine and Public Health, School of Medicine, University of California, 3855 Health Sciences Drive, Room 3077, La Jolla, CA 92093-0901 USA

**Keywords:** Weight loss, Nuts, Satiety, Cardiovascular disease risk factors, Blood pressure

## Abstract

**Background:**

Dietary strategies that help patients adhere to a weight reduction diet may increase the likelihood of weight loss maintenance and improved long-term health outcomes. Regular nut consumption has been associated with better weight management and less adiposity. The objective of this study was to compare the effects of a walnut-enriched reduced-energy diet to a standard reduced-energy-density diet on weight, cardiovascular disease risk factors, and satiety.

**Methods:**

Overweight and obese men and women (*n* = 100) were randomly assigned to a standard reduced-energy-density diet or a walnut-enriched (15% of energy) reduced-energy diet in the context of a behavioral weight loss intervention. Measurements were obtained at baseline and 3- and 6-month clinic visits. Participants rated hunger, fullness and anticipated prospective consumption at 3 time points during the intervention. Body measurements, blood pressure, physical activity, lipids, tocopherols and fatty acids were analyzed using repeated measures mixed models.

**Results:**

Both study groups reduced body weight, body mass index and waist circumference (time effect *p* < 0.001 for each). Change in weight was −9.4 (0.9)% vs. -8.9 (0.7)% (mean [SE]), for the standard vs. walnut-enriched diet groups, respectively. Systolic blood pressure decreased in both groups at 3 months, but only the walnut-enriched diet group maintained a lower systolic blood pressure at 6 months. The walnut-enriched diet group, but not the standard reduced-energy-density diet group, reduced total cholesterol and low-density lipoprotein cholesterol (LDL-C) at 6 months, from 203 to 194 mg/dL and 121 to 112 mg/dL, respectively (*p* < 0.05). Self-reported satiety was similar in the groups.

**Conclusions:**

These findings provide further evidence that a walnut-enriched reduced-energy diet can promote weight loss that is comparable to a standard reduced-energy-density diet in the context of a behavioral weight loss intervention. Although weight loss in response to both dietary strategies was associated with improvements in cardiovascular disease risk factors, the walnut-enriched diet promoted more favorable effects on LDL-C and systolic blood pressure.

**Trial registration:**

The trial is registered at (NCT02501889).

## Introduction

Current guidelines for the management of overweight and obesity recommend prescribing a reduced-energy diet as a primary treatment intervention to promote weight loss, as part of a comprehensive lifestyle intervention, and conclude that a variety of dietary approaches can produce weight loss [1]. However, dietary patterns, specific foods, and macronutrient composition may differentially affect metabolic factors, satiety, and the postprandial gastrointestinal peptide response that could affect hunger and appetite [2, 3]. Dietary strategies that help patients reduce energy intake and adhere to a reduced-energy diet may increase the likelihood of improved long-term health outcomes and reduced risk for obesity-related conditions and diseases.

In several large cohorts and a few clinical trials, a dietary pattern that includes regular nut consumption has been associated with less weight gain in adulthood and a lower degree of adiposity [4–11]. In a few previous studies, the effects of consuming almonds, pistachios, walnuts and peanuts on weight change and cardiovascular disease risk factors in the context of a weight loss intervention have been examined, with mixed results [12–18]. A proposed mechanism for the favorable effect of nuts on weight control is that they promote increased satiety, resulting in a compensatory reduction in total energy intake [4, 5]. Feelings of satiety, fullness, and hunger following walnut consumption has been examined in only a few previous studies. In those studies, acute postprandial peptide response and early phase satiety was observed to be similar following a meal with or without walnuts, although increased satiety and fullness were found on days 3 and 4 following a walnut-containing meal [19, 20]. Measuring responses over the long-term would better model the observational studies that have linked regular nut consumption with lower adiposity and better weight control.

In the present study, we compared the effects of a walnut-enriched reduced-energy diet to a reduced-energy-density diet, which has been suggested to be a useful dietary strategy to promote reduced energy intake without compromising meal satiety [21]. The primary objective of this study was to compare the effects of a walnut-enriched reduced-energy diet to a standard reduced-energy-density diet on body weight and cardiovascular disease risk factors in a sample of overweight and obese adults in an intensive 6-month weight loss intervention. A secondary objective was to examine whether there is a differential response in satiety- and appetite-related ratings scales in association with a walnut-enriched reduced-energy diet and a reduced-energy-density diet among the participants in this weight-loss study.

## Methods

### Subjects

**Fig. 1:**
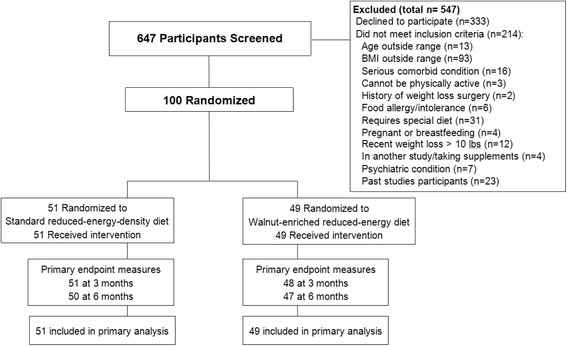
Flow chart for study participants

One hundred non-diabetic overweight and obese men and women were randomized from a screened sample of 647 (Fig. 1). To be included in the study, participants had to meet the following criteria: Aged 21 years and older, body mass index (BMI) between 27 and 40 kg/m^2^; willing and able to participate in clinic visits, group sessions, and telephone and internet communications; able to provide data through questionnaires and telephone; willing to maintain contact with investigators for 6 months; willing to allow blood collections; no known allergy to tree nuts; and capable of performing a simple test for assessing cardiopulmonary fitness. Exclusion criteria were any of the following: Inability to participate in physical activity due to severe disability; history or presence of a comorbid diseases where diet modification and increased physical activity may be contraindicated; self-reported pregnancy or breastfeeding or planning a pregnancy within the next year; currently involved in another diet intervention study or weight loss program; and having a history or presence of a significant psychiatric disorder or any condition that would interfere with participation in the trial. The University of California, San Diego (UCSD), institutional review board approved the study protocol, and all participants provided written informed consent.

Prior to enrollment, potential participants were screened for diabetes and considered ineligible with a fasting blood glucose ≥125 mg/dL. At screening and recruitment, the ability to participate in moderate intensity physical activity was assessed by questionnaire, a standard procedure for screening participants for community-based weight loss programs of this nature. Participants were additionally asked to report all prescription medications and were asked if they had ever been told by a doctor that they had high blood cholesterol. Once enrolled, participants were randomly assigned to one of the two study arms using a sequence stratified by age (≤52 vs. >52 years) and BMI (≤33 vs. >33 kg/m^2^).

### Intervention

All participants were provided a detailed diet prescription in an individual counseling session with a dietitian, in which a caloric deficit was set based on the participant’s goals, and a sample meal plan was developed according to study arm and participant food preferences. The overall goal of the dietary guidance was to promote a reduction in energy intake, aiming for a 500- to 1000-kcal/day deficit relative to expenditure. All participants had follow-up contact with the dietitian by telephone or email a minimum of every 1–2 weeks for additional support and to reinforce adherence throughout the intervention.

Participants assigned to the standard reduced-energy-density diet arm were provided diet plans that emphasized lower energy density food choices such as vegetables, fruit and whole grains, as well as lean protein sources and reduced-fat dairy foods, with macronutrient composition within current guidelines (https://www.choosemyplate.gov/MyPlate). Participants assigned to this study group were asked to refrain from eating any nuts (and products containing them) for the duration of the study.

Participants assigned to the walnut-enriched reduced-energy study group were instructed to consume an average of 42 g (1.5 oz) of walnuts/day for diet prescriptions that were ≥1500 kcal/day, or 28 g (1 oz) of walnuts/ for diet prescriptions <1500 kcal/day, all within their energy-reduced diet plan (thus, walnuts provided approximately 15% of total energy intake). Participants were provided meal and snack suggestions and recipes to facilitate adherence, and the nuts were distributed to participants assigned to that group on a weekly basis for 12 weeks and then biweekly for the remainder of the study. Also, participants were queried about walnut consumption for the previous week when the walnuts were distributed, and adherence was recorded.

Use of a Web-based planning and tracking program that enabled tracking kilocalories was encouraged. All participants were provided a scale and were asked to weigh themselves daily and to record their progress. An activity tracker was provided and participants were asked to gradually build up to a minimum of 10,000 steps per day within the first month and then to maintain or increase that level of lifestyle activity. An additional daily exercise goal was an average of at least 60 min/day of purposeful aerobic activity at a moderate level of intensity. Strength training 2–3 times/week also was encouraged. Tools such as measuring cups, small exercise equipment, and videos were provided to encourage adherence.

In addition to individualized diet prescription and counseling, all participants were assigned to a series of closed group sessions (weekly for 12 weeks, then biweekly), based on a semi-structured cognitive-behavioral weight loss intervention. Briefly, strategies discussed included: Planning and tracking meals and exercise; environmental control; realistic goal-setting; triggers to eating and ways to deal with them; problem-solving; dealing with negative thoughts; promoting self-efficacy through goal accomplishments and other strategies; self-nurturing; dealing with lapses; and addressing body image concerns.

### Measurements

Study data were collected and managed using a Research Electronic Data Capture (REDcap) database hosted at UCSD [22]. At baseline and 3- and 6-month follow-up data collection clinic visits, weight, height (baseline only), waist circumference, and blood pressure were measured, and a fasting (≥6 h) blood sample and questionnaires were collected. Systolic and diastolic blood pressure was averaged from two sitting blood pressure measurements. The 3-min step test, which measures heart rate during the first 30 s of recovery from stepping, was used to assess cardiopulmonary fitness. This test has high reliability and is sensitive to change [23].

Physical activity was estimated using the Godin Leisure-Time Exercise Questionnaire, a validated self-report measure of physical activity that has been widely used in previous research [24]. This questionnaire assesses weekly hours of moderate and strenuous physical activity. These data were compared with current recommendations for physical activity in adults, which are 150 min weekly of moderate physical activity, or 75 min weekly of strenuous physical activity, or a combination of these [25].

Participants were asked to rate general (rather than meal-specific) satiation by using a visual analog scale (VAS), an approach which has been shown to have validity, reliability, and reproducibility [26]. Similar to other studies in which satiety and satiation over time (rather than meal-specific) have been assessed [19], participants were asked to complete these scales before lunch and dinner meals at three time points during the 6 months of active participation (weeks 1, 6, and 13). Specifically, subjects were asked to rate their satiety by answering three questions. Each of the questions was completed by the participant and transferred by staff (blinded to study arm assignment) to a REDCap (Vanderbilt University, Nashville, TN, USA) file database, with a 100 mm horizontal line anchored at either end, so that answers can be quantified on a continuous scale. The questions are: “How hungry do you feel?” with anchor values ranging from “I have never been more hungry” (scored as 0) to “I am not hungry at all” (scored as 100); “How full do you feel?”, with anchor values ranging from “Not at all full” (scored as 0) to “Totally full” (scored as 100); and “How much do you think you could eat now?” with anchor values ranging from “Nothing at all” (scored as 0) to “A lot” (scored as 100).

### Laboratory measures

Laboratory measurements were conducted with plasma samples that had been frozen at -80^ο^C after blood collection and processing. Total cholesterol, triglycerides, and high-density lipoprotein cholesterol (HDL-C) were measured by Arup Laboratories (Salt Lake City, UT, USA) using enzymatic methods. The coefficient of variation (CV) for human serum for cholesterol at 76.2 mg/dL and 276 mg/dL is 1.6% and 1.4%, respectively; for triglycerides at 104 mg/dL and 261 mg/dL is 1.9% and 1.8%, respectively; and for HDL-C at 46.4 mg/dL and 80.4 mg/dL is 0.6% and 0.7%, respectively. Low-density lipoprotein cholesterol (LDL-C) values were calculated by the Friedewald equation [27].

Tocopherols and fatty acids were measured as dietary biomarkers because we anticipated that the walnut-enriched diet group could have different circulating concentrations compared to participants in the standard diet arm, reflecting differential intake of these dietary constituents due to regular walnut consumption. The detection and quantification of plasma tocopherols was accomplished by high performance liquid chromatography, using fluorescent detection at a wavelength of 295 nm excitation and 325 nm emission. Tocopherols were quantified by peak height using a standard curve prepared in bovine serum matrix from pure external compounds. Additionally, pooled in-house quality control samples were analyzed concurrently with batches of study samples, together with other commercially available reference samples, to monitor accuracy and precision. Also, the laboratory participates in the National Institute of Standards and Technology quality assurance program.

Red blood cell (RBC) fatty acids were measured by OmegaQuant Laboratories (Sioux Falls, SD, USA) by gas chromatography (GC) with flame ionization detection. GC was carried out using a GC2010 Gas Chromatograph (Shimadzu Corporation, Columbia, MD, USA) equipped with a SP2560, 100-m fused silica capillary column (0.25 mm internal diameter, 0.2 um film thickness; Supelco, Bellefonte, PA, USA). Fatty acids were identified by comparison with a standard mixture of fatty acids characteristic of RBCs (GLC OQ-A, NuCheck Prep, Elysian, MN, USA) which was also used to determine individual fatty acid calibration curves. Fatty acid composition was expressed as a percent of total identified fatty acids.

### Statistical analysis

Demographic characteristics were compared at baseline between groups using chi-square tests for categorical variables and t-tests for continuous variables. Body measurements (weight, BMI, waist circumference), blood pressure, physical activity, lipids, tocopherols and fatty acids were analyzed using repeated measures mixed models assuming unstructured covariance. Change in an indicator of adiposity between groups (weight change as a percentage of initial weight) was also analyzed. Study time, diet group, and the group by time interaction were modeled as fixed effects in each model. Variables that were skewed were log transformed in analysis.

Lipid concentrations were examined by sex to assess significant differences at baseline. We tested to see which of the lipids changed between baseline and 6 months, and if a significant change was observed, we performed multivariate analysis to identify predictors of such a lipid change.

Power analysis for our sample size was based on published literature for nut consumption in a weight loss intervention [15–17]. Significance was set at alpha = 0.05. All statistical analysis was performed using the SAS software version 9.4 for Windows (SAS Institute Inc., Cary, North Carolina, USA).

## Results

During the course of the study, 3 participants dropped out (one in the standard diet group and 2 in the walnut-enriched diet group). Overall compliance with prescribed walnut consumption in that study arm was 98%; review of monitoring records indicated that of the 47 participants, 43 reported consuming 97–100%, 2 reported consuming 92–96%, and 2 reported consuming 67–69% of the walnuts prescribed during the study.

As shown in Table 1, the randomized study groups did not differ by sex, age, education, or race/ethnicity. Both groups demonstrated a reduction in body weight, BMI, and waist circumference (time effect *p* < 0.001 for each) during the course of the study, and the two diet groups did not differ in degree of weight lost, with no significant group by time interactions, as shown in Table 2. Both groups decreased their systolic blood pressure at 3 months, but only those in the walnut-enriched diet group maintained a lower systolic blood pressure at 6 months compared to baseline (Table 3). Participants in both study groups also decreased their diastolic blood pressure at 3 and 6 months, and increased their physical activity (*p* < 0.001 for each). There was no significant group by time interaction observed in the blood pressure or physical activity models (Table 3). Cardiopulmonary fitness, as indicated by the step test recovery heart rate, improved in both study groups.

**Table 1:** Characteristics of study participants in the weight reduction intervention

	Standard reduced-energy-density diet (*n* = 51)	Walnut-enriched reduced-energy diet (*n* = 49)	*p* (between groups) *
Sex (N [%])			0.53
Female	27 (53%)	31 (63%)	
Male	24 (47%)	18 (37%)	
Age (years), mean (SE)	52.2 (1.6)	53.3 (1.4)	0.63
Education (years), mean (SE)	16.1 (0.3)	16.2 (0.3)	0.88
Race/ethnicity (%)			0.84
Non-Hispanic white	73	73	
Hispanic/Latino	14	18	
African-American	6	2	
Asian-American	2	2	
Mixed/other	6	4	

*p values are from chi-square tests (categorical variables), or t-tests (continuous variables)

**Table 2:** Body measurements of study participants in the weight reduction intervention

	Standard reduced- energy-density diet		Walnut-enriched reduced-energy diet		*p* (between groups)
	n	Mean (SE)	n	Mean (SE)	
Body weight, kg ^a^					
Baseline	51	90.9 (1.8)	49	91.1 (2.3)	0.96
3 Months	51	84.7 (1.8)	48	85.9 (2.3)	0.70
6 Months	50	82.1 (2.0)	47	82.4 (2.2)	0.92
Body mass index, kg/m^2 a^					
Baseline	51	32.4 (0.4)	49	32.4 (0.5)	0.96
3 Months	51	30.3 (0.5)	48	30.6 (0.5)	0.63
6 Months	50	29.4 (0.6)	47	29.6 (0.5)	0.77
Weight change, kg					
3 Months	51	−6.0 (0.6)	48	−5.5 (0.5)	0.51
6 Months	50	−8.5 (0.9)	47	−7.9 (0.6)	0.58
% Weight change					
3 Months	51	−6.6 (0.6)	48	−6.1 (0.6)	0.53
6 Months	50	−9.4 (0.9)	47	−8.9 (0.7)	0.63
Waist circumference, cm ^a^					
Baseline	51	109.9 (1.2)	49	111.5 (1.6)	0.42
3 Months	51	101.7 (1.3)	48	104.6 (1.6)	0.16
6 Months	50	98.9 (1.4)	47	100.7 (1.5)	0.39

^a^ Body weight, body mass index, and waist circumference showed a significant time effect compared with baseline, *p* < 0.001 for each variable, in both study groups at each follow-up point

**Table 3:** Blood pressure and physical activity variables for study participants in the weight reduction intervention

	Standard reduced-energy-density diet		Walnut-enriched reduced-energy diet		*p* (between groups)
	n	Mean(SE)	n	Mean(SE)	
Systolic blood pressure, mm Hg					
Baseline	51	123 (2)	49	124 (3)	0.77
3 Months	49	117 (2) *	48	116 (2) *	0.73
6 Months	49	119 (2)	46	118 (2) *	0.68
Diastolic blood pressure, mm Hg					
Baseline	51	82 (1)	49	82 (2)	0.72
3 Months	49	77 (1) *	48	76 (1) *	0.57
6 Months	49	78 (2) *	46	77 (1) *	0.70
Moderate/strenuous physical activity, minutes/week					
Baseline	51	120 (22)	49	133 (18)	0.53
3 Months	51	328 (31) *	48	337 (33) *	0.84
6 Months	49	351 (31) *	47	321 (29) *	0.48
% Meeting physical activity recommendations					
Baseline	51	25	49	45	0.04
3 Months	51	78	48	79	0.93
6 Months	47	85	47	81	0.58
Step test, heart rate/30s					
Baseline	51	57 (2)	49	60 (2)	0.14
3 Months	47	47 (1) *	47	49 (1) *	0.33
6 Months	47	45 (1)*	45	47 (1) *	0.18

* Different from baseline within group, *p* < 0.01 for each

Participants assigned to the walnut-enriched diet group, but not the standard reduced-energy-density diet group, had a reduction in total cholesterol concentration at 6 months, from 203 to 194 mg/dL (*p* = 0.04), as shown in Table 4. Triglycerides decreased in the standard diet group at 3 months and in both groups at 6 months, which decreased an average of 22 mg/dL from 128 to 106 (*p* < 0.01 in log-transformed analysis). HDL-C did not change significantly between baseline and 6 months in either of the diet groups. In a subgroup analysis among the 21 men in the study, those assigned to the walnut-rich diet group had lower HDL-C levels (42 [10] vs. 50 [7] mg/dL [mean (SD)]) than those assigned to the standard reduced-energy-density diet at baseline (*p* = 0.05) and at 3 months, 41(9) vs 54 (13) mg/dL (*p* = 0.02) (data not shown). By 6 months, the men assigned to the walnut-enriched diet group had increased their HDL-C to 49 (18) mg/dL, and those in the standard reduced-energy-density diet group had also increased HDL-C to 59 (13) mg/dL (data not shown). Although 27% of the cohort reported having been told by a doctor that they had high cholesterol, only 10% of the cohort reported taking prescription medications to lower lipids.

**Table 4:** Biological measures of study participants in the weight reduction intervention

	Standard reduced-energy-density diet	Walnut-enriched reduced-energy diet	*p* (between groups)	*p* (group x time interaction)
	Mean(SE)	Mean(SE)		
Cholesterol, mg/dL				0.84
Baseline	200 (5)	203 (6)	0.76	
3 Months	199 (5)	198 (5)	0.95	
6 Months	194 (6)	194 (6) ^a^	0.91	
Triglycerides, mg/dL				0.50
Baseline	130 (10)	123 (7)	0.55	
3 Months	110 (8) ^a^	115 (9)	0.66	
6 Months	109 (9) ^a^	103 (6) ^a^	0.61	
HDL cholesterol, mg/dL				0.08
Baseline	58 (2)	59 (2)	0.70	
3 Months	60 (2)	58 (2)	0.37	
6 Months	60 (2)	61 (2)	0.94	
LDL Cholesterol, mg/dL				0.60
Baseline	116 (4)	121 (5)	0.42	
3 Months	116 (5)	116 (4)	0.80	
6 Months	112 (5)	112 (5) ^a^	0.96	
Alpha-tocopherol, μmol/L				0.96
Baseline	30.5 (1.3)	30.8 (1.0)	0.83	
3 Months	30.0 (1.1)	30.3 (1.2)	0.72	
6 Months	31.6 (1.2)	32.2 (1.3)	0.84	
Beta-tocopherol, μmol/L				0.70
Baseline	0.33 (0.02)	0.33 (0.01)	0.38	
3 Months	0.38 (0.01) ^a^	0.27 (0.01) ^a^	0.38	
6 Months	0.28 (0.01) ^a^	0.26 (0.01) ^a^	0.99	
Gamma-tocopherol, μmol/L				0.48
Baseline	4.23 (0.29)	3.99 (0.27)	0.55	
3 Months	4.04 (0.31)	4.13 (0.20)	0.82	
6 Months	4.08 (0.33)	4.30 (0.29)	0.74	
Delta-tocopherol, μmol/L				0.98
Baseline	0.11 (0.01)	0.11 (0.01)	0.82	
3 Months	0.10 (0.01)	0.10 (0.01)	0.88	
6 Months	0.09 (0.01) ^a^	0.08 (0.01) ^a^	0.76	
Linoleic acid, %				<0.001
Baseline	0.111 (0.002)	0.110 (0.002)	0.77	
3 Months	0.104 (0.002) ^a^	0.111 (0.002)	0.004	
6 Months	0.107 (0.002) ^a^	0.112 (0.001) ^a^	0.01	
Alpha-linolenic acid, %				<0.001
Baseline	0.00122 (0.00006)	0.00118 (0.00004)	0.57	
3 Months	0.00105 (0.00006) ^a^	0.00147 (0.00005) ^a^	<0.001	
6 Months	0.00118 (0.00005)	0.00158 (0.00007) ^a^	<0.001	

^a^ Different from baseline within group, *p* < 0.05

The overall change (in both groups combined) in total cholesterol at 6 months was −7 mg/dL and for triglycerides was −20 mg/dL. A multivariate model for change in triglycerides did not show that diet group assignment, weight loss, age, sex, or level of physical activity were significantly associated; however, a model for change in total cholesterol showed that weight change and age were significantly associated. In the multivariate model for change in cholesterol at 6 months, R-squared was 0.17 and the two factors significantly associated were age (*p* = 0.002) and weight change (*p* = 0.02). Diet, baseline BMI, baseline physical activity, and change in physical activity were not significantly related to cholesterol change. Participants >50 years of age decreased their cholesterol by 2 mg/dL compared with a decrease of 19 mg/dL for subjects younger than 50 years of age. Those who lost ≥5% of initial weight decreased their cholesterol by an average of 13 mg/dL compared with an increase in cholesterol of 18 mg/dL in subjects who did not lose at least 5% of initial body weight.

As shown in Table 4, we did not observe changes in alpha- or gamma-tocopherol, which are the major tocopherols in the plasma, and only minor changes in beta- and delta-tocopherol concentrations. Also, we observed increased concentrations of alpha-linolenic acid and linoleic acid in the walnut-enriched diet group over the study period, but not in the standard reduced-energy-density diet group (Table 4).

Self-reported satiety was similar across the study in the diet groups (Table 5). Feelings of hunger decreased and fullness was greater at week 12 than week 1 in the standard reduced-energy-density diet group (*p* < 0.05). Fullness was lower in the walnut-rich diet arm at week 12 (*p* = 0.04).

**Table 5:** Self-reported satiety (on a 100-point visual analog scale where Hunger is scored 0 = very hungry, 100 = not hungry at all; Fullness is scored 0 = not full, 100 = full; Quantity is scored 0 = nothing, 100 = a lot) in the weight reduction intervention

	Standard reduced-energy-density diet, mean(SEM)		Walnut-enriched reduced-energy diet, mean(SEM)	
	Lunch	Dinner	Lunch	Dinner
Hunger				
Week 1	43 (3)	40 (4)	49 (3)	40 (3)
Week 6	50 (4)	43 (4)	42 (4)	45 (4)
Week 12	53 (5) ^a^	49 (4) ^a^	44 (4)	44 (4)
Fullness				
Week 1	44(4)	48 (5)	51(4)	51(5)
Week 6	53 (5)	53 (42)	58 (4)	52 (5)
Week 12	52 (6) ^a^	61 (5) ^a^	48 (4) ^b^	49 (4) ^b^
Quantity				
Week 1	44(4)	50 (4)	49 (3)	55(4)
Week 6	49 (4)	53 (4)	48 (4)	47 (4)
Week 12	41 (4)	38 (5)	44 (3)	49 (4)

^a^ Both hunger and fullness were greater at week 12 than week 1 in the standard reduced-energy-density diet group (*p* < 0.05)

^b^ At week 12, fullness was lower in the walnut-rich diet arm than in the standard reduced-energy-density diet group (*p* = 0.04)

## Discussion

Findings from this study provide further evidence that a walnut-enriched reduced-energy diet can promote weight loss that is comparable to a standard reduced-energy-density diet in the context of a behavioral weight loss intervention. Although weight loss in response to both dietary strategies was associated with improvements in lipids and blood pressure, the walnut-enriched diet promoted more favorable effects on some cardiovascular disease risk factors, such as LDL-C and systolic blood pressure.

Previous studies that have examined the effect of prescribing regular nut consumption on weight change in a weight loss intervention have had mixed results. Two studies found more weight loss in association with almond consumption (at doses of 50–84 g/day for 3 months) compared with controls [12, 16], while a study examining the effect of a similar amount of almonds over a longer time frame (18 months) did not observe more weight loss compared to controls [15]. In a study that examined the effects of prescribing peanuts (16% of energy), weight loss was similar to controls, although the peanut-containing study arm had more favorable effects on cardiovascular disease risk factors [13]. Providing a daily snack of pistachios (53 g/day) vs. pretzels promoted a greater reduction of BMI and plasma triglyceride concentration but only a trend for a difference in body weight change in another study [14]. In a 12-month intervention study aimed to promote weight loss and healthy lifestyle, prescribing 30 g/day walnuts was associated with greater weight loss and improved diet quality compared to providing general dietary advice during the 3-month intensive phase of the intervention, although these differences were not evident at study end [17].

We recently examined the effects of a walnut-rich or higher-monounsaturated fat diet vs. a lower-fat diet prescription on weight loss and selected lipids and biomarkers in the context of a 12-month behavioral weight loss program [18, 28]. Participants were stratified by insulin resistance status to allow examination of whether insulin resistance might be associated with differential response to diet composition. Similar to the present study, we observed that prescribing walnuts was associated with weight loss that was comparable to a standard lower fat diet, but better than a higher fat, lower carbohydrate diet without walnuts with regard to biomarker response [18].

In addition to promoting a similar degree of weight loss, we observed similar self-reported satiety in response to a walnut-enriched reduced-energy diet and a reduced-energy-density diet, that has been proposed to promote reduced energy intake without compromising meal satiety [21]. Notably, walnuts are very high in energy density, but when consumed as a component of a reduced-energy diet, this strategy may help to promote adherence to restricted total energy intake.

The effects of tree nuts on blood lipids and several other cardiovascular disease risk factors were recently examined in a systematic review and meta-analysis [29], as well as in an earlier pooled analysis [30], and our observations of lower cholesterol and LDL-C in response to walnut consumption are in agreement with their conclusions. Across the 61 trials that met the eligibility criteria for the meta-analysis, that study found an average reduction of −4.7 and −4.8 mg/dL for total cholesterol and LDL-C, respectively, per one ounce/day serving of tree nuts in interventions ranging from 3 to 26 weeks [29]. Results of the present study, in which we observed this walnut-specific effect to be even greater in the context of a weight loss intervention, add to the evidence base. We also observed the effect to be modulated by age and degree of weight loss, with a greater reduction in cholesterol in younger individuals (<50 years) and those with greater weight loss (≥5% of initial weight). Previous meta-analyses of the effects of nut consumption on blood pressure are not in agreement, with one of them concluding that there are no significant effects [29] and another showing a reduction in systolic blood pressure in participants without type 2 diabetes [31] as observed in the present study.

Walnuts are rich in gamma-tocopherol and polyunsaturated fatty acids, particularly alpha-linolenic and linoleic fatty acids [32]. In previous studies, an increase in gamma-tocopherol concentration has been observed in participants who were prescribed daily walnut consumption [33, 34]. In our previous trial that prescribed walnuts in a weight loss intervention [18, 35], we observed that walnut prescription minimized the reduction in plasma gamma-tocopherol that occurs in association with reduced energy intake and weight loss, as was observed in the present study. The increase in RBC alpha-linolenic and linoleic fatty acid concentrations in those assigned to the walnut-enriched reduced-energy study arm, and the differences across diet groups, is consistent with previous walnut feeding and walnut-rich diet interventions [18, 33, 36]. These changes in dietary biomarkers also provide strong support for the self-reported high level of adherence in participants instructed to consume walnuts daily in the present study. Notably, replacing saturated fats with polyunsaturated fats has been consistently associated with reduced risk for cardiovascular disease [37, 38].

This study has some strengths and limitations. A strength is the heterogeneity of the study sample, which included both men and women and participants across racial/ethnic groups. Also, the retention rate was very high, which is not typical of weight loss intervention studies, and this reduces ambiguity in drawing inferences from this study. A limitation of the study is the lack of detailed information about dietary intake. We encouraged study participants to self-monitor dietary intake as a component of the behavioral strategies to promote weight control, but we did not collect detailed dietary data in an effort to minimize subject burden. Because this was a sample of free-living individuals, some variability in adherence to the prescribed diet is likely. However, the weight loss demonstrated by study participants suggests that most were consuming a reduced-energy diet, and the RBC fatty acid biomarker is indicative of good compliance by participants in the walnut-enriched diet group.

## Conclusions

In conclusion, findings from this study provide further evidence that a walnut-enriched reduced-energy diet can promote weight loss that is comparable to a standard reduced-energy-density diet in the context of a behavioral weight loss intervention. Weight loss in response to both of these dietary strategies was associated with improvements in lipids and blood pressure, although the walnut-enriched diet promoted more favorable effects on LDL-C and systolic blood pressure.

## Abbreviations

BMI: Body mass index
CV: Coefficient of variation
GC: Gas chromatography
HDL-C: High-density lipoprotein cholesterol
LDL-C: Low-density lipoprotein
RBC: Red blood cell
REDcap: Research Electronic Data Capture
SE: Standard error
UCSD: University of California, San Diego
VAS: Visual analog scale
